# Assessing noise sources at synchrotron infrared ports

**DOI:** 10.1107/S0909049511041884

**Published:** 2011-11-25

**Authors:** Ph. Lerch, P. Dumas, T. Schilcher, A. Nadji, A. Luedeke, N. Hubert, L. Cassinari, M. Boege, J.-C. Denard, L. Stingelin, L. Nadolski, T. Garvey, S. Albert, Ch. Gough, M. Quack, J. Wambach, M. Dehler, J.-M. Filhol

**Affiliations:** a Swiss Light Source, Paul Scherrer Institut, CH-5232 Villigen, Switzerland; b Synchrotron SOLEIL, Saint-Aubin, F-91192 Gif-sur-Yvette, France; c Laboratorium für Physikalische Chemie, ETH-Zürich, CH-8093 Zürich, Switzerland

**Keywords:** noise, FTIR, spectroscopy

## Abstract

Today, the vast majority of electron storage rings delivering synchrotron radiation for general user operation offer a dedicated infrared port. There is growing interest expressed by various scientific communities to exploit the mid-IR emission in microspectroscopy, as well as the far infrared (also called THz) range for spectroscopy. Compared with a thermal (laboratory-based source), IR synchrotron radiation sources offer enhanced brilliance of about two to three orders of magnitude in the mid-IR energy range, and enhanced flux and brilliance in the far-IR energy range. Synchrotron radiation also has a unique combination of a broad wavelength band together with a well defined time structure. Thermal sources (globar, mercury filament) have excellent stability. Because the sampling rate of a typical IR Fourier-transform spectroscopy experiment is in the kHz range (depending on the bandwidth of the detector), instabilities of various origins present in synchrotron radiation sources play a crucial role. Noise recordings at two different IR ports located at the Swiss Light Source and SOLEIL (France), under conditions relevant to real experiments, are discussed. The lowest electron beam fluctuations detectable in IR spectra have been quantified and are shown to be much smaller than what is routinely recorded by beam-position monitors.

## Abstract 2

Low-frequency noise present in the electron and photon beams of two comparable storage rings, SOLEIL and SLS, are carefully compared in the context of IR spectroscopy using the Fourier transform technique.

## 1. Introduction

Most of the experiments performed at the infrared ports of synchrotron radiation facilities involve the use of Fourier transform infrared (FTIR) spectrometers [see, for example, Griffiths & de Haseth (1986 ▶) and Quack & Merkt (2011 ▶)]. The main advantage of using FTIR techniques rather than a dispersive method is the ability to measure the IR response of a sample across a rather wide wavelength range by sampling all wavelengths simultaneously (multiplex advantage). In order to achieve this, the entire spectral information is coded, with the use of a Michelson-like interferometer, into AC signals. The typical frequencies of these AC signals range between a few tens of Hz to a few tens of kHz, depending of the velocity of the moving mirror of the interferometer and the bandwidth of the detector. Strictly speaking, the actual quantity of interest measured with a Fourier-transform spectrometer is the transmitted light as a function of the distance travelled by the moving mirror in the Michelson arrangement, the ‘interferogram’. When operated in the continuous scan mode, the resulting interferogram is digitized at the zero crossings of the interference pattern produced by monochromatic light (typically He-Ne 633 nm or 15.798 cm^−1^) which propagates through the instrument along the same optical pathlength as the IR light under investigation. A sample placed in the optical path introduces a change in transmitted signal, that we call a variation in optical retardation. In order to increase the signal-to-noise ratio, the measurements are repeated several times and the averaged interferogram is then processed with a Fourier-transform algorithm. The transmission spectrum of the sample is the ratio of the spectra measured with and without sample in the optical path.

An IR synchrotron radiation source has a theoretical brilliance advantage of about two to three orders of magnitude over a thermal (laboratory-based) source. This advantage is well exploited at all IR beamlines, despite appearing rather modest when compared with the brilliance advantage offered by a synchrotron radiation X-ray source compared with a standard X-ray tube, for example. However, in order to fully exploit this advantage to the benefit of IR spectroscopy, it should not be spoiled by significant instabilities present in the electron beam and/or along the rather long optical path of typical beamlines (Reffner *et al.*, 1995 ▶; Carr *et al.*, 1995*a* ▶,*b* ▶; Lobo *et al.*, 1999 ▶; Carr, 2001 ▶). Any perturbation of the source position and/or intensity, as well as variations of the optical étendue along the optical path owing to vibration of one or other optical element, will limit the ability of the instrument to detect changes of optical retardation owing to the sample only. In order to put things into practical context, let us now assume that the amplitude of the IR source coupled to a FTIR bench is modulated by 1% at a constant frequency of, say, 630 Hz and that the moving mirror of the interferometer (also called scanner) runs at 0.316 cm s^−1^ (which corresponds to a 10 kHz sampling rate of the interferogram). The entire photon beam carries this additional 630 Hz ‘noise’ which gets stubbornly coded into the interferogram. Considering the wavelength of the He-Ne laser (633 nm corresponds to 15798 cm^−1^) used to track the zero-crossings of the interferogram, this noise appears, after Fourier transformation, as a spectral artifact in the important mid-IR region at (15798 cm^−1^ × 630/10000) ≃ 1000 cm^−1^ which corresponds to a 10 µm wavelength.

**Figure 1:**
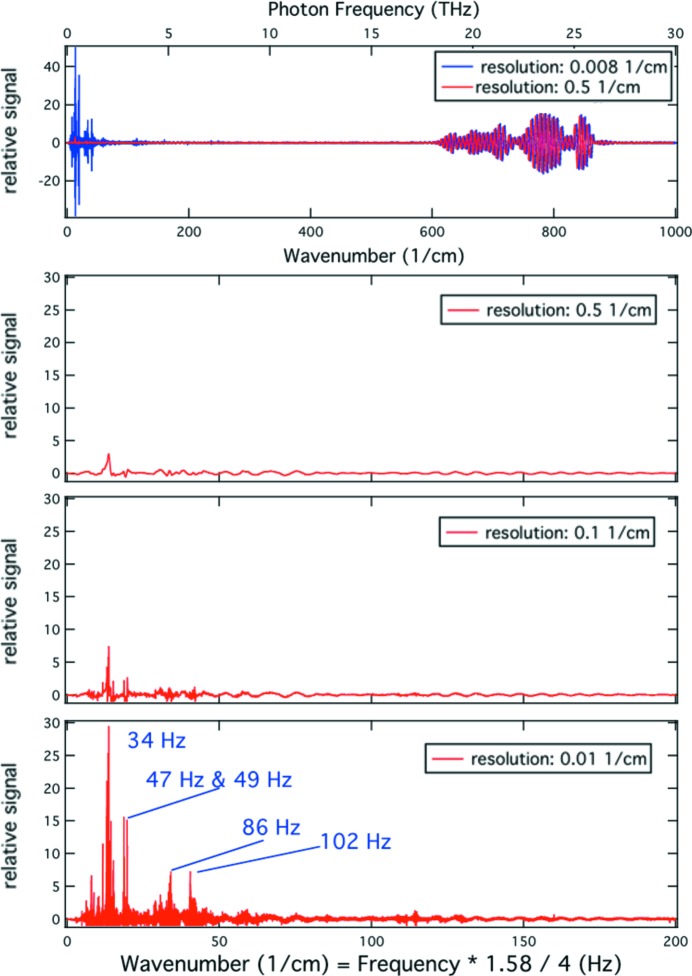
Top panel: entire spectrum of benzole measured at the SLS in a long-path cell. Absorption actually occurs between 600 and 900 cm^−1^. The three following panels show low-frequency noise outside the region of interest measured at different resolutions. The residual low-frequency noise is important at the highest resolution. Scanner rate: 40 kHz.

Fig. 1 ▶ gives the opportunity to appreciate the problem. The top panel shows the FTIR spectrum for an organic substance measured in its gas form. The experiment was carried out in a long-path cell using the Swiss Light Source (SLS) synchrotron source, a high-resolution FTIR bench, a KBr beam splitter and a liquid-nitrogen-cooled IR detector that was insensitive to light below 500 cm^−1^. The spectral region of scientific interest lies between 600 and 900 cm^−1^ whereas the region below 200 cm^−1^ (no IR light detected) is dominated by spectral features owing to noise. The following three panels illustrate, as expected, the fact that noise increases with increasing resolving power. The spectral features identified at 34, 47, 49, 86 and 102 Hz need to be suppressed if experiments are to be carried out in this range. For that particular measurement (top panel), however, low-frequency noise was of limited importance and valid high-resolution data could be obtained (Albert *et al.*, 2011 ▶).

For all IR synchrotron facilities it is of utmost importance to identify (if possible beforehand) and harness intrinsic and external noise sources. Intrinsic noise sources are those perturbations (size and position) carried by the electron beam itself, whereas external noise sources are mainly due to minute mechanical instabilities acting on storage ring components and/or beamline elements that affect the optical path and étendue. In some cases, extrinsic perturbations (mechanical, electrical, magnetic) which are transferred to the storage ring can affect the electron beam itself and are further transferred to the photon beam. The various instability sources have been carefully considered in the design and construction of third-generation storage rings. The settlement, buildings and storage ring components were designed with utmost care in order to maintain the electron beam stability required to operate in the top-up mode. Complex dynamic feedback systems (Hubert *et al.*, 2009 ▶; Schilcher *et al.*, 2004 ▶) that use beam-position monitor (BPM) (Hubert *et al.*, 2007 ▶; Dehler *et al.*, 1999 ▶) data as input have been designed and installed in order to maintain and monitor the short- (typically below 1 µm vertical and horizontal up to 100 Hz) and long-term stability of the electron beam orbit. Despite all these efforts, however, noise still limits the performance of IR beamlines and must be investigated. Noise in the RF system of the storage ring can induce significant artifacts (Byrd, 1999 ▶). Variations of the source position, for example, are efficiently harnessed by an active mirror steering system which has tremendously helped in increasing performances of several beamlines worldwide (Scarvie *et al.*, 2004 ▶). However, this system cannot compensate for source size variations. Perturbations owing to line frequency variations have been addressed with a line trigger system (Bosch & Julian, 2002 ▶).

In order to investigate various noise sources and quantify their impact on IR spectroscopic research, we measured noise across a wide frequency range, present at two different IR ports, SLS and SOLEIL. These two storage rings have quite similar electron energies, sizes and performances. In order to complement noise measurements observed at the IR ports, we also recorded low-frequency noise spectra acquired by BPM and XBPM systems in the same storage ring sector. The purpose of this work is not to rank the facilities according to their performances, but rather to emphasize that, even for third-generation machines, noise remains a problem of concern for IR studies, even if less severe than noise observed with earlier generation storage rings. The diagnostic aspects discussed in this work will be helpful as well in finding remedies to remaining problems in the future.

## 2. Sources and beamlines

The IR port at the Swiss Light Source, X01DC, collects 60 (H) mrad × 40 (V) mrad dipole radiation from a bending magnet (electron energy of 2.4 GeV, magnetic field of 1.4 T). The SOLEIL port collects 78 (H) mrad by 20 (V) mrad of edge and bending-magnet radiation (electron energy of 2.75 GeV, magnetic field of 1.72 T). See Table 1 ▶ for some machine parameters.

At the SLS, the first optical element is a water-cooled plane mirror with a central horizontal slot of height 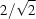
 mm, placed approximately 820 mm from the ideal source ‘point’. The extracted synchrotron radiation light is transported to the experimental area using three segments of 1:1 optics. The first segment includes one focusing element, a *f*/*D* = *N* ≃ 3 toroidal Al mirror. Several moveable UHV-compatible IR transparent windows (diamond, silicon and BaF2) separate the UHV of the storage ring from the beamline high-vacuum volume near the first focal point. The second and third transport segments include one focusing element (Al-coated glass mirrors) with *N* ≃ 3. The beamline is terminated with a KBr or diamond window. There are three equivalent ports, named F3A, F3B and F3C, where instrumentation can be coupled. In order to couple fast-Fourier-transform spectrometers, a divergence matching optics is inserted between the F3 points of the beamline and the input focus of the optical bench. Details on F3C and beamline optics can be found by Albert *et al.* (2011 ▶) and Carroll *et al.* (2011 ▶), respectively.

There are two independent IR beamlines at SOLEIL named AILES (Roy *et al.*, 2006 ▶) and SMIS (Dumas *et al.*, 2006 ▶). They have identical collection angles and source types (edge radiation plus constant field). We carried out this work at the SMIS beamline. The extraction mirror is a plane Au-coated Al mirror, located at 1.21 m from the source point, with a central horizontal slot of height 2.56 mm. The beamline is composed of three segments. The first one includes the extracting plane mirror and a pair of toroidal mirrors which focus the beam through a 20 mm CVD diamond window, located at 11.02 m from the source point. The second segment includes two plane mirrors which steer the beam to a mirror housing located outside the radioprotection hutch. Then the beam is split into two branches using a combination of flat and toroidal mirrors. For each branch the beam is focused through a KBr or CVD diamond window, before being collimated and directed into the IR spectrometer. In this work, we use the constant field branch of the SMIS beamline.

## 3. Sensitivity

The FTIR bench coupled to an IR port could readily be used to assess performances and study noise issues. In this work, in order to obtain a fair comparison between facilities, we performed noise measurements at the two facilities using the same detector. A four-quadrant (4Q) light detector (InGaAs PIN photodiode, four-quadrant element, 2 mm diameter, Hamamatsu G6849; spectral response is observed to be broader than values indicated in the specification sheet) is placed near a focal point before the entrance of the spectrometer. The position of the detector was adjusted to minimize the signal recorded in the horizontal and vertical directions. The electrical output of the four quadrants are wired so as to deliver amplitude differences in the horizontal and vertical directions, as well as the sum signal. This allows one to disentangle vertical and horizontal fluctuations. The total signal was used to normalize the data. Spectra are expressed as normalized power spectral density (PSD). Visible-wavelength neutral density filters were used when necessary to prevent detector saturation owing to intense visible synchrotron light.

**Figure 2:**
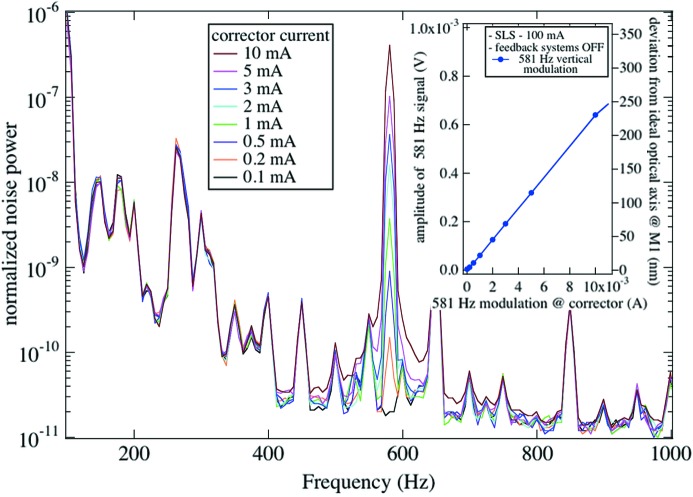
Spectral distribution of light power measured in the vicinity of a focal point at the Swiss Light Source. The strong feature at 581 Hz is the response owing to the vertical angular modulation of the electron beam imposed by a corrector magnet. Feedback systems are off.

In order to determine the sensitivity of a typical IR beamline to source position fluctuations, we performed the following simple experiment. The SLS storage ring was filled with approximately 100 mA of current, and the fast and slow orbit feedback systems were switched off. A corrector magnet was fed with an AC current at a frequency of 581 Hz (this would correspond, in the IR spectra, to 1000 cm^−1^ for a sampling rate of 10 kHz). The amplitude of the corrector current was reduced from 10 mA down to almost 0 mA in several steps while the spectral distribution of light power was recorded. An equivalent experiment was performed at SOLEIL with 22 mA stored current and a modulation frequency of 86 Hz. Figs. 2 ▶ and 3 ▶ show the normalized spectral distribution of light power from which the external modulation at 581 Hz (SLS) and 86 Hz (SOLEIL) can be nicely isolated. As expected, the light intensity varies linearly with the amplitude of the corrector current. The other spectral features that are visible are due to noise.

A modulation current of 10 mA in the corrector magnet of the SLS results in a peak of relative noise power of 10^−6^. The peak is some three to four orders of magnitude above the background at that frequency and corresponds to a variation of light intensity of 0.1%. With the precise knowledge of the lattice dynamics parameters and the deviations measured at the BPMs placed upstream and downstream of the bending magnet of that sector of the storage ring, we can evaluate the corresponding vertical deviation from the ideal optical axis anywhere in the vicinity of the bending magnet. A particularly interesting case is the corresponding vertical deviation of the photon beam from its ideally centred optical axis through the slotted extraction mirror. The angular change of the electron trajectory of 0.12 mrad A^−1^ imposed by the corrector magnet translates to a vertical deviation of 23 µm A^−1^. The right-hand-side scales of the insets of Figs. 2 ▶ and 3 ▶ illustrate the actual sensitivity of the beam position that can be observed with a simple 4Q detector.

**Figure 4:**
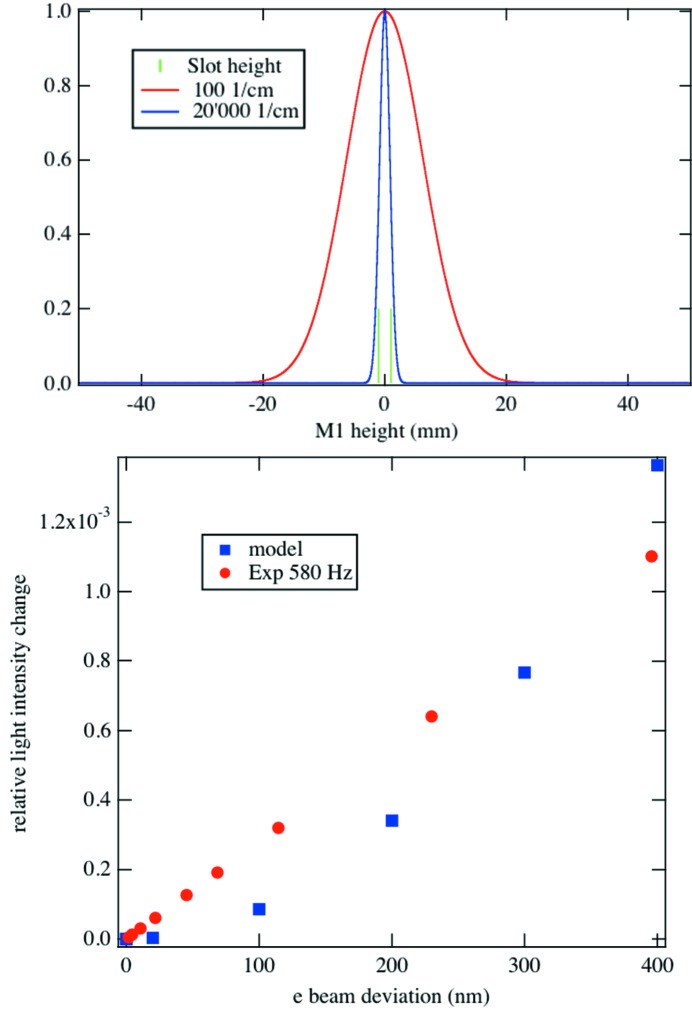
Top: vertical light intensity profile at two photon energies. The size of the slot in M1 is depicted. Bottom: measured and calculated relative flux change reflected by a 2 mm slotted M1 mirror.

The sensitivity to vertical beam motion can be estimated using the following crude approach that captures the essentials. Let ϕ be the natural (vertical) divergence angle of the synchrotron radiation, ϕ = 1.66(λ/ρ)^1/3^, where λ and ρ are the radiation wavelength and the bending radius, respectively. ρ(λ) is used to estimate the width σ_*y*_ of a Gaussian profile of the spectral density of light hitting M1, the first optical element of the beamline. Thus, σ_*y*_ is wavelength dependent: σ_*y*_ = σ_*y*_(λ). Let us assume a uniform horizontal distribution of light. Because M1 (of both facilities) has a slit, a fraction of the radiation gets clipped. We estimate the radiation reflected by the active part of M1 and compute the relative difference captured in the case where the beam has been subjected to a small deviation δ, 
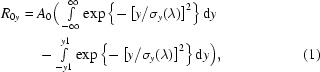


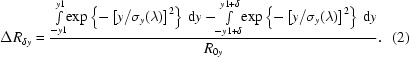
In (1), 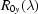 represents the spectral distribution of photons reflected by M1, taking into account the loss owing to the slot. Equation (2) is then used to compute the relative flux difference, 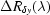, owing to a beam kick of amplitude δ. At the top of Fig. 4 ▶ the width of the simplified vertical light profile varies with photon energy, reflecting the trend imposed by the wavelength dependence of the vertical divergence angle. At much higher photon energy, when the beam profile width at M1 becomes narrower than the slot width, the relative change 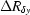 decreases to zero. In the range of interest for IR work, however, the sensitivity to vertical beam deviations increases with increasing photon energy.

In our beam wobbling experiments the entire photon beam carries the modulation frequency (581 Hz at SLS and 86 Hz at SOLEIL). To estimate the relative signal change owing to beam modulation, we must integrate 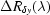 taking into account the wavelength response range of the 4Q detector as well as the wavelength dependence of the vertical divergence of the synchrotron radiation. The result is shown in Fig. 4 ▶. Given its simplicity, we do not expect this model to predict any better than the correct order of magnitude. Using a more involved approach, that calculates the exact initial wavefront and propagates it through the entire beamline, with several known small modifications of the optical étendue (beam kick amplitude), is possible using the package *SRW* for example, but turns out to be a much more involved endeavor. Relations (1) and (2) are practical and help to carry out noise-reduction measures at IR beamlines.

## 4. Noise

Let us now compare the normalized noise present in the audio frequency range when both storage rings are operated with closed feedback loops.

**Figure 5:**
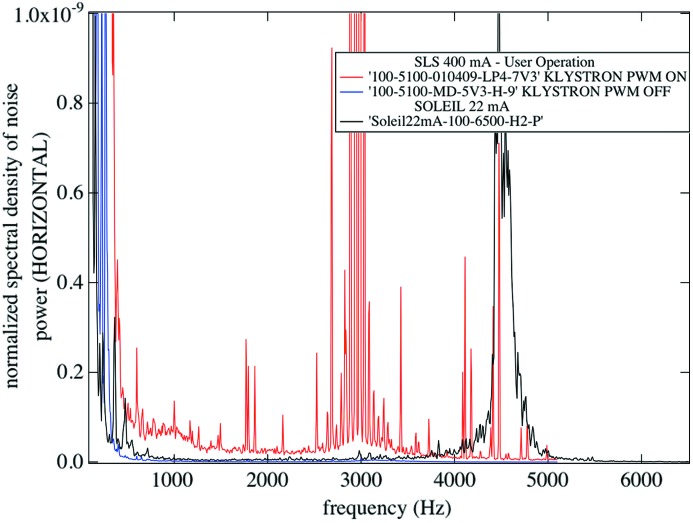
Spectral distribution of horizontal normalized noise power measured in the vicinity of a focal point at the SLS and SOLEIL. Noise at both ports is comparable, provided proper settings of the RF power supplies at SLS are chosen.

Fig. 5 ▶ illustrates the situation at both storage rings. In regular operation, the SLS exhibits a fair number of significant intensity excursions beween 1 and 5 kHz, with a strong feature peaking around 3 kHz. The SLS synchrotron resonance is expected around 7 kHz at 400 mA of stored current. At SOLEIL the main feature visible in this frequency range is its synchrotron resonance around 4.7 kHz. In addition, between 500 Hz and 3 kHz, the background noise present at SLS is larger. It is of importance to find the exact cause of these intensity variations since the strong 3 kHz features will show up around 4700 cm^−1^ assuming a 10 kHz sampling rate and, even worse, around 1000 cm^−1^ if the sampling rate is increased to 40 kHz. Without any solution to get rid of that noise, this situation will cancel a fair amount of the brilliance advantage provided by the synchrotron. Dedicated scrutiny on a variety of sub-systems allowed us to clearly isolate and suppress the most important source of noise that dominates in Fig. 5 ▶ and was limiting the performances of IR work at the SLS storage ring.

The possible noise contribution from the bending-magnet supply was verified by adding a separate precision DCCT system (http://www.hitecsms.com/) with a small signal bandwidth of 100 kHz to the dipole supply cable. The measured signal was rather featureless in the frequency domain, in comparison with the signals measured at the IR port, and was not considered further.

The possible noise contribution from RF phase noise was verifed. The SLS RF 500 MHz distribution system uses two IRF2040 generators running in phase lock with each other. By monitoring the DC FM signal on an oscilloscope, the system phase noise is known. The DC FM signal was ±1 mV peak, corresponding to ±3.5 Hz peak frequency deviation. However, this signal was synchronized to the main frequency, with a large component 600 Hz, and without a random component; given the low input level to the oscilloscope, this signal is most likely an artifact and the true phase noise should be much lower. In addition, if the phase noise power is ascribed equally to the two sources, this value should also be reduced by a factor of 0.707 to ±2.5 Hz peak. An external sine-wave phase/frequency modulation was then introduced to the 500 MHz sources, and the resulting spectrum was monitored at the IR port. Arbitrary modulation levels were used, simply enough to give a spectral line magnitude comparable with the unwanted features observed at the IR port. The injected level necessary was typically ±30 Hz peak frequency deviation, far more than the intrinsic source deviation. RF phase noise was not considered further.

Excess noise was identified to originate from a pulse width modulation (PWM) system, regulating the voltage of the RF power supply. At the SLS, each of the four accelerating cavities are fed by high-power klystrons with about 100 kW RF power. The high-voltage power supplies of these klystrons are pulse step modulators (PSMs). They consists of a set of 68 smaller power supplies (modules) connected in series, operated with a PWM frequency of 100 kHz and rotation of the modules for equal power loading. The PSM system generates side-bands in the audio frequency range on the 500 MHz carrier, which translate into variations of the electron energy. In turn, these variations affect the trajectory at a point of dispersion; the source gets ‘wobbled’. We assessed the presence of that horizontal noise at two other beamlines, in the UV and soft X-ray energy ranges, using appropriate detectors. This observation was the proof that the perturbations were in the electron beam and not confined at the level of the beamline.

The lowest-frequency component generated by the PSM is about 1.47 kHz and corresponds to the PWM frequency divided by the number of modules. However, the most pronounced peak found in the IR spectrum corresponds to about 2.94 kHz, the second harmonic of this frequency.

To get rid of this problem, the SLS PWM system was switched off, and to our great satisfaction the situation improves by three orders of magnitude around 3 kHz, and by approximately one order of magnitude below and above that frequency. Regular operation of the SLS storage ring without the PWM system is entirely to the benefit of IR work. In this situation the relative noise levels at the two facilities are hardly distinguishable (see Fig. 5 ▶ for PWM off). There are indications that some images recorded with segmented X-ray detectors benefit from the new mode of operation as well, and work is in progress to quantify this effect.

The RF system installed at SOLEIL uses superconducting cavities together with solid-state amplifier technology. First, the cavities have high quality factors, so that out-of-band artifacts are very efficiently suppressed. Second, solid-state amplifiers use conventional switched power supplies. The use of this technology results in optimal performances.

**Figure 6:**
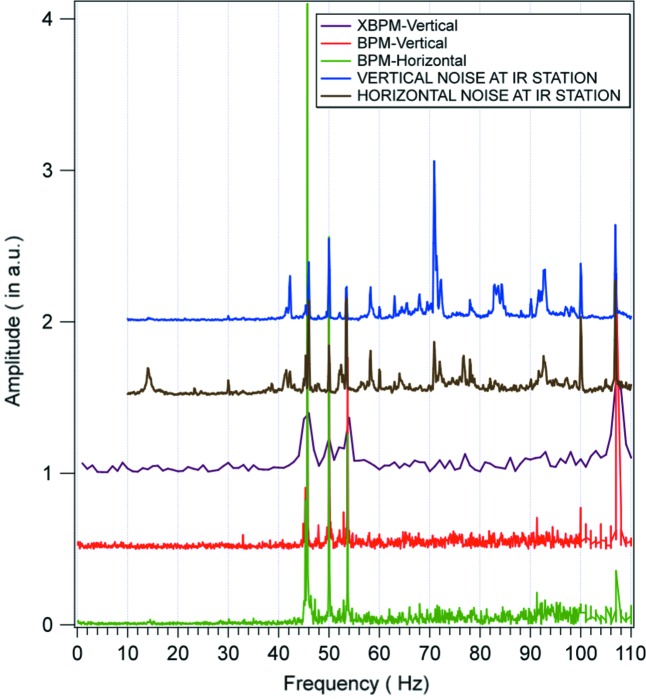
Noise recording (horizontal and vertical) at one SOLEIL IR endstation compared with the BPM and XBPM recording in the 0–110 Hz frequency range.

At SOLEIL we recorded the frequency spectra at the electron- and X-BPMs while recording noise at the IR port. Fig. 6 ▶ shows that modes at 45, 50, 54 and 108 Hz are registered by both electronic and photon diagnostic systems. These modes, present in the electron beam already, have been clearly identified as coming from variable magnetic fields at those frequencies induced by fans that cool ceramic vacuum vessels. They are going to be suppressed in the near future. On the spectra recorded with the 4Q detectors, in addition to the above-mentioned modes, some extra features are visible, and are most likely due to mechanical instabilities of one or the other optical element of the beamline itself. In this low-frequency range, such noise sources must be identified and if possible minimized in order to allow for very high resolution investigations in the far-IR energy range.

## 5. Impact of induced beam motion on IR spectra

**Figure 7:**
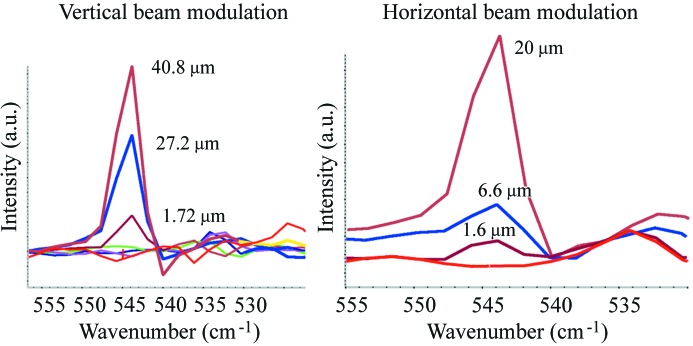
Artifacts observed in the FTIR spectrum for horizontal and vertical variations of the position of the electron beam. The SOLEIL beam was modulated at 86 Hz and the artifacts are detected at 544 cm^−1^, for a scanner rate of 2.5 kHz, and with a spectral resolution of 4 cm^−1^.

**Figure 8:**
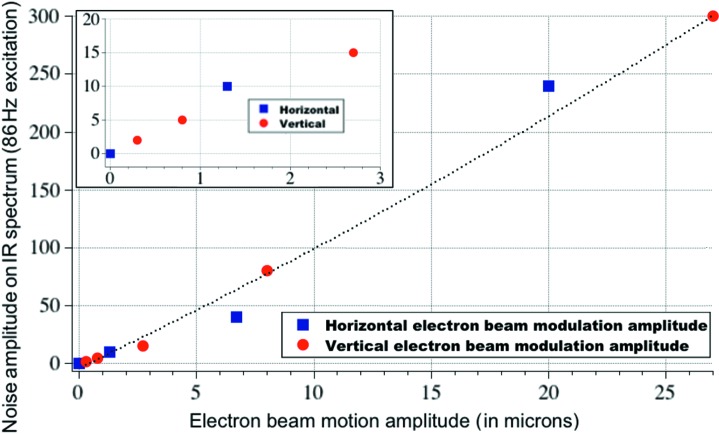
Artifact amplitude as a function of electron beam deviation. Results were obtained at SOLEIL with a beam wobbling frequency of 86 Hz.

The question to what extent the electron beam instabilities will become detrimental to the quality of an IR spectrum was addressed at the IR port at SOLEIL. In this study we generated a perturbation at 86 Hz by a corrector magnet, and, by varying its amplitude, we recorded the amplitude of the spectral features visible in the IR spectra. This amplitude depends on the details of the coupling between the IR port and the illumination and collection optics of each microspectrometer, as well as on the true bandwidth and acquisition parameters. Fig. 7 ▶ shows the interesting portion of IR spectra, recorded with an electron beam current of 10 mA and a mirror velocity of the interferometer corresponding to a 2.5 kHz sampling rate. As expected, the amplitude of the additional feature visible in the IR spectra decreases with the deviation imposed on the electron beam orbit. The noise pick-up becomes barely observable below 1 µm of electron beam deviation. The beam was wobbled horizontally and vertically with equivalent amplitudes. We observe that IR spectra (in the mid- to far-IR range) are equally sensitive to each direction of the electron beam deviation. A comment is in order here. In §3 we discussed sensitivity to ‘vertical’ beam motion, knowing that the geometry of the extraction optics, namely the horizontal slot in M1, is the first (because in the design) reason for sensitivity along the vertical axis. In this section we observe that horizontal and vertical beam motions result in equivalent artifacts, at the end of the entire beamline–instrument–detector system. This is due to the fact that the FTIR bench transforms any light intensity variation (in particular those owing to a change in optical étendue occurring along the optical path) into spectral artifacts. Fig. 8 ▶ shows the amplitude of the spectral artifact as a function of electron beam deviation. IR beamlines that perform with a typical spectral resolution of 4 cm^−1^ require a beam stability of the order of, or better than, 1 µm. This very tight requirement is achieved at third-generation machines so that no additional dynamical beam steering between the IR port and the spectrometer input is required.

**Figure 9:**
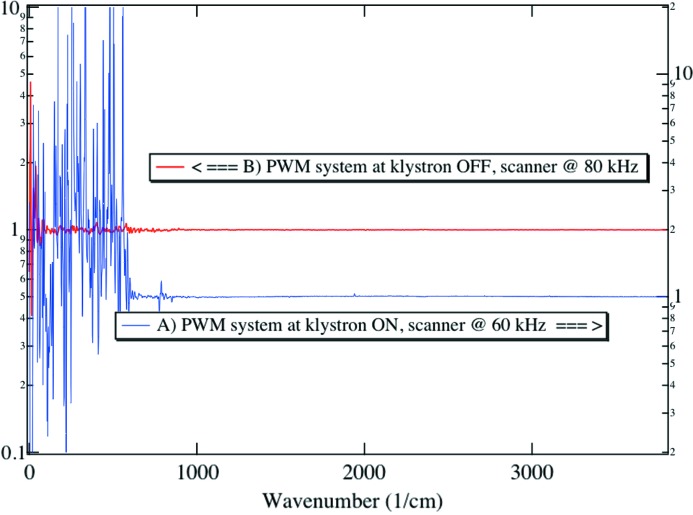
Ratio of two consecutive IR spectra (average of 100 spectra, resolution 4 cm^−1^) measured through a 10 µm-diameter pinhole placed in the focus of an IR microscope equipped with a pair of X15 Schwarzchild optics. (*A*) RF sub-system causing excess noise turned on. (*B*) RF sub-system turned off.

In order to emphasize the practical benefit on spectral quality obtained at the SLS IR port when the noise owing to the RF power supply sub-system is suppressed, we show in Fig. 9 ▶ the ratio of two consecutive measurements, with and without noise source. Light was coupled into the FTIR spectrometer, itself coupled to the IR microscope, and focused through a 10 µm-diameter pinhole. This arrangement mimics a practical transmission experiment. In a perfect system the ratio of two consecutive spectra should yield a perfect flat line with amplitude 1. Spectroscopists call this a 100% line. In spectral regions where the detector receives less light, the amplitude of noise increases. When the RF sub-system causing excess noise is in operation (curve A) the signal-to-noise ratio calculated by integrating any spectral range exceeds the values obtained from the same integration on curve B. Degradation is strong in the frequency range that corresponds to the 3 kHz features discussed in the previous section, as well as at low frequency. Moreover, the artifact measured near 770 cm^−1^ on curve A using a sampling rate of 60 kHz shifts to lower wavenumber when sampled at 80 kHz.

## 6. Conclusion

In this work we carefully compared low-frequency noise present in the electron and photon beams of two comparable storage rings, SOLEIL and SLS, in the context of IR spectroscopy using the Fourier transform technique. It turns out that IR ports are valuable beam diagnostic tools which can, with a moderate instrumental investment, provide better sensitivity than electron- and X-BPMs. We confirmed that the beam stabilization tools (BPM and feedback systems) installed at two third-generation storage rings are capable of stabilizing the beam position below 1 µm in a bending magnet, and that this performance matches the requirements for IR spectroscopy. In the case of the SLS, it was found that a pulse width modulation system used to regulate the high-voltage power supplies of the klystrons was generating too much noise (kHz range, outside the active band of the feedback systems) to enable IR work. Turning off this system appears to be a cost-effective solution, and to date no negative side effect (except perhaps a slight degradation of RF amplifier efficiency) has been reported.

At both ports we observe in the low-frequency range (say, below 100 Hz) several discrete contributions to the total noise. For the case of the SLS, the main contributions are observed at 34, 47, 49 and 102 Hz. The cause is not entirely clarified, but there are strong indications that these perturbations are of mechanical origin. The 34 Hz mode for instance is mostly a horizontal beam motion, visible at the IR port as well at another X-ray photon port. Interestingly, this mode is also observed using a seismic sensor placed on the bending magnet, and a mirror chamber to probe the vertical and horizontal axes. Mechanical energy at 34 Hz along the horizontal axis dominates (and is present at another beamline) and may coincide with the resonance frequency of a mechanical element that has a weakness along the same direction. The amplitude of this mode is constant in time. On the other hand the amplitude of the 49 Hz mode is not constant with time, and exhibits a slowly varying period of 8–10 min. These perturbations impose optical beam deflections larger in amplitude than those corresponding to a 1 µm electron beam deflection. If high-resolution work is envisaged in this wavenumber range (34 Hz gives 53 cm^−1^ at 10 kHz scanner rate and 102 Hz turns into 161 cm^−1^), the exact source of this noise must be identified and cancelled.

We carried out a careful noise analysis of the IR beamline environment at the SLS. On one occasion there was the opportunity to measure mechanical and optical noise through the beamline when the synchrotron was not in operation and the building essentially ‘powered off’ (with the exception of all the sublimation pumps, controls computers, sensors and the linac was in standby) for a couple of hours. A sensitive seismic sensor was placed on the bending magnet and measured the spectral distribution of acceleration along the vertical gravitational axis. With an optical laser coupled through a quartz window into the back of the bending magnet and steered along the optical axis through the entire beamline, we measured the vertical and horizontal low-frequency noise power carried by the laser beam in the same fashion as when noise in the synchrotron beam was measured.

**Figure 10:**
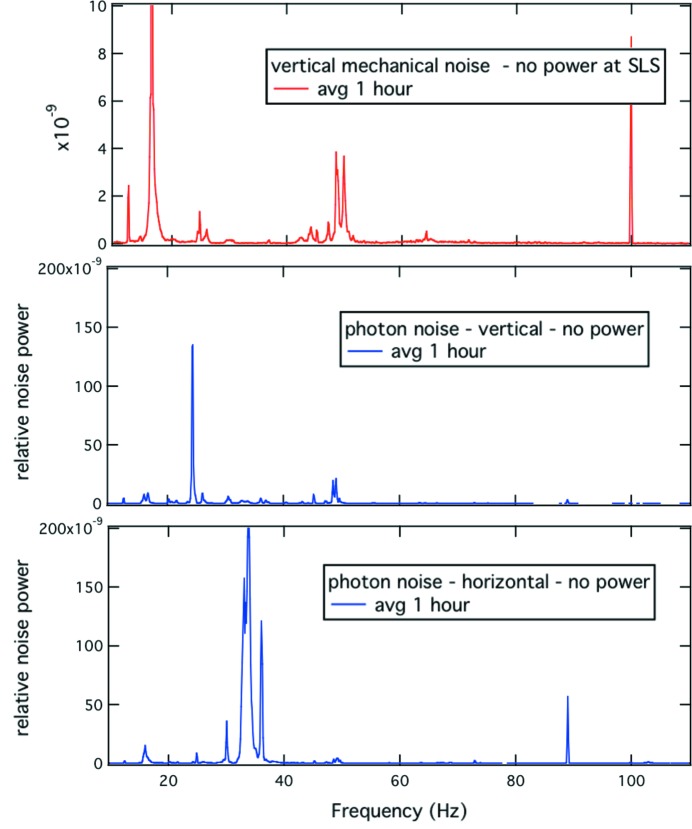
For these measurements the SLS power is essentially turned off (except linac and sublimation pumps, controls sensors and computers). Top panel: mechanical noise, along the vertical axis measured with a seismic sensor placed on the bending magnet. Middle and bottom panels: vertical and horizontal normalized optical noise power density, respectively, measured with a 4Q sensor near a focal point, using a laser beam aligned through the main optical axis of the beamline.

Without entering into a detailed description of these extensive measurements we summarize some of the findings. Data in Fig. 10 ▶ represent the quietest situation that can presently be achieved. The 34 Hz mode we observe during regular synchrotron operation is absent from the spectrum of the vertical mechanical noise recorded on the bending magnet but clearly dominant in the horizontal optical data (bottom panel) measured with the laser through the beamline, the facility being essentially at rest (SLS ‘power off’). In the mechanical data there is a dominant contribution at 16.6 Hz as well as a genuine contribution of energy at 30 Hz. There is no horizontal mechanical noise data available for the same ideal (but unrealistic) situation. However, a significant 34 Hz horizontal mechanical contribution is observed when all the systems (ventilation, cooling water *etc.*) are operational.

The ‘breathing’ mode at 49 Hz (distinct from 50 Hz) observed during regular operation in the vertical optical data is present in the vertical mechanical ‘powered off’ data. When the facility mains are turned back on (not shown), the amplitude of that mode increases significantly. This breathing mode has clearly a remote mechanical origin. An electrical artifact degrading the measurement apparatus can be excluded because seismic and optical data correlate (two-channel time domain correlation). Moreover, for the optical data, de-coupling is naturally provided by the 4Q sensor that acts as a transducer. We observe this mode in both situations, SLS powered on and off with two different sources of mains. As of writing, the precise origin of these two low-frequency contributions to noise remains unknown.

## Figures and Tables

**Figure 3:**
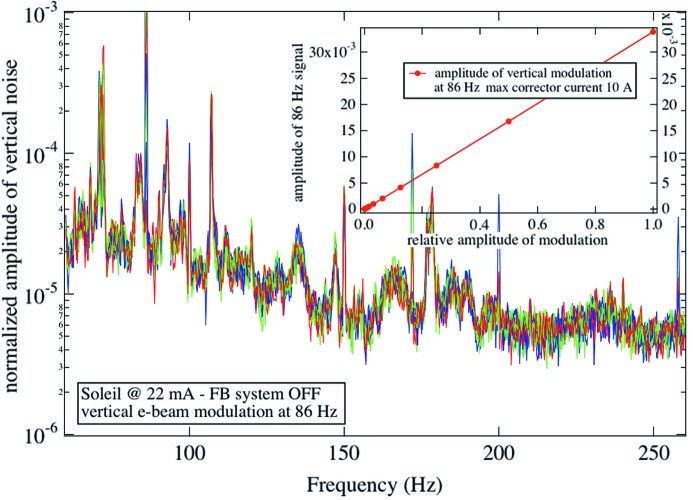
Spectral distribution of light intensity measured in the vicinity of a focal point at SOLEIL. The strong feature at 86 Hz is the response owing to the vertical angular modulation of the electron beam imposed by a corrector magnet. Feedback systems are off.

**Table 1:** Machine parameters ν_*x*_ and ν_*z*_ are the betatron tunes in the 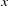 and 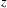 directions, respectively. H and V are (1 to 150 Hz) cumulated noise values for the position of the electron beam expressed at the dipole of each IR port and measured with a BPM excluded from the feedback loop and situated at another location of the lattice.

	Energy (GeV)	Radius (m)	Field (T)	ν_*x*_/ν_*z*_	Emittance (nm rad)	Energy spread	H/V noise (µm r.m.s.)	Slot (mrad)
SOLEIL	2.75	5.36	1.72	18.20/10.3	3.74	1.016 × 10^−3^	0.4/1.2	2.1
SLS	2.4	5.75	1.4	20.43/8.73	5	9 × 10^−4^	0.6/1.1	1.72
